# Reactive oxygen species facilitate the EDH response in arterioles by potentiating intracellular endothelial Ca^2+^ release

**DOI:** 10.1016/j.freeradbiomed.2016.06.010

**Published:** 2016-08

**Authors:** James Chidgey, Paul A. Fraser, Philip I. Aaronson

**Affiliations:** a King's College London, Faculty of Life Sciences and Medicine, Division of Asthma, Allergy & Lung Biology, London, United Kingdom; b King's College London, Faculty of Life Sciences and Medicine, Cardiovascular Division, London, United Kingdom

**Keywords:** AACOCF_3_, arachidonyl trifluoromethyl ketone, AUDA, 12-(3-adamantan-1-yl-ureido)dodecanoic acid, CP85, Na-1-ethylsulfoxide-2-methyl-3-hyroxypyridin-4-one, CP94, 1,2-diethyl-3-hyroxypyridin-4-one, CPA, Cyclopiazonic Acid, DFO, Desferrioxamine, EC, endothelial cell, EDH, endothelial dependent hyperpolarization, EDHF, EDH factor, EDHR, EDH response, EETs, epoxyeicosatrienoic acids, EEZE, 4, 15-epoxyeicosa-5(Z)-enoic acid, sEH, epoxide hydrolase, EGTA, ethylene glycol-bis(β-aminoethyl ether)-N,N,N′,N′-tetraacetic acid, HEPES, 4-(2-hydroxyethyl)-1-piperazineethanesulfonic acid, IBTx, Iberiotoxin, IL-1⍰, interleukin-1β, IP_3_R, IP_3_ receptor, L-NAME, N(G)-nitro-l-arginine methyl ester, L-NNA, Nω-Nitro-l-arginine, NO, nitric oxide, ODYA, 17-octadecynoic acid, PE, phenylephrine, PGI_2_, prostacyclin, PKG, protein kinase G, PLA_2_, phospholipase A_2_, cPLA_2_, cytoplasmic PLA_2_, PLC, phospholipase C, PPOH, 2-(2-propynyloxy)-benzenehexanoic acid, PSS, physiological salt solution, ROS, reactive oxygen species, SERCA, sarcoplasmic reticulum Ca^2+^-ATPase, SOD, superoxide dismutase, S&C, SOD and catalase, VSMC, vascular smooth muscle cell, Endothelium dependent hyperpolarization, Arteriole, Ca^2+^, Reactive oxygen species, Vasodilation, H_2_O_2_

## Abstract

There is abundant evidence that H_2_O_2_ can act as an endothelium-derived hyperpolarizing factor in the resistance vasculature. However, whilst scavenging H_2_O_2_ can abolish endothelial dependent hyperpolarization (EDH) and the associated vascular relaxation in some arteries, EDH-dependent vasorelaxation can often be mimicked only by using relatively high concentrations of H_2_O_2_. We have examined the role of H_2_O_2_ in EDH-dependent vasodilatation by simultaneously measuring vascular diameter and changes in endothelial cell (EC) [Ca^2+^]_i_ during the application of H_2_O_2_ or carbachol, which triggers EDH. Carbachol (10 µM) induced dilatation of phenylephrine-preconstricted rat cremaster arterioles was largely (73%) preserved in the presence of indomethacin (3 µM) and l-NAME (300 µM). This residual NO- and prostacyclin-independent dilatation was reduced by 89% upon addition of apamin (0.5 µM) and TRAM-34 (10 µM), and by 74% when an extracellular ROS scavenging mixture of SOD and catalase (S&C; 100 U ml^−1^ each) was present. S&C also reduced the carbachol-induced EC [Ca^2+^]_i_ increase by 74%. When applied in Ca^2+^-free external medium, carbachol caused a transient increase in EC [Ca^2+^]_i_. This was reduced by catalase, and was enhanced when 1 µM H_2_O_2_ was present in the bath. H_2_O_2_ -induced dilatation, which occurred only at concentrations ≥100 µM, was reduced by a blocking antibody to TRPM2, which had no effect on carbachol-induced responses. Similarly, iberotoxin and Rp-8bromo cGMP reduced the vasodilatation induced by H_2_O_2_, but not by carbachol. Inhibiting PLC, PLA_2_ or CYP450 2C9 each greatly reduced the carbachol-induced increase in EC [Ca^2+^]_i_ and vasodilatation, but adding 10 µM H_2_O_2_ during PLA_2_ or CYP450 2C9 inhibition completely restored both responses. The nature of the effective ROS species was investigated by using Fe^2+^ chelators to block the formation of ∙OH. A cell permeant chelator was able to inhibit EC Ca^2+^ store release, but cell impermeant chelators reduced both the vasodilatation and EC Ca^2+^ influx, implying that ∙OH is required for these responses. The results indicate that rather than mediating EDH by acting directly on smooth muscle, H_2_O_2_ promotes EDH by acting within EC to enhance Ca^2+^ release.

## Highlights

• We recorded carbachol- evoked endothelial cell (EC) [Ca^2+^]_i_ rises in rat arterioles.
• Ca^2+^ rises were reduced by catalase, cPLA_2_ and CYP29C blockers, and iron chelators.
• EC Ca^2+^ release was potentiated by 30 pM IL-1β or 1 μM exogenous H_2_O_2_.
• H_2_O_2_ and carbachol evoked vasodilation, but by dissimilar mechanisms.
• H_2_O_2_ promotes hyperpolarization-dependent dilatation by increasing EC Ca^2+^ release.

## Graphical abstract

**Figure:**
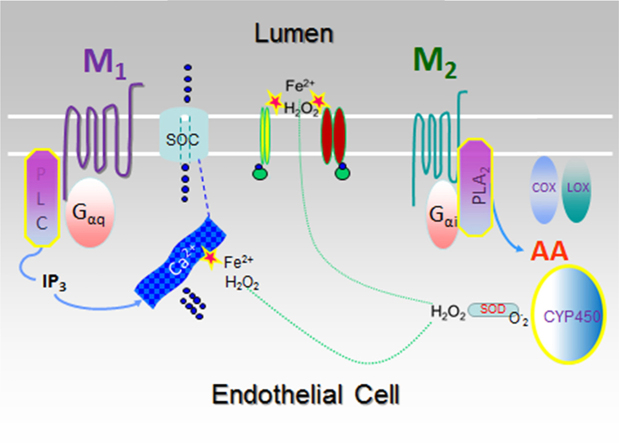

## 1 Introduction

Endothelium-dependent hyperpolarization (EDH) plays an important role in the vasodilatation of small arteries and arterioles induced by diverse stimuli including locally released factors, blood flow, and low pH [1]. EDH was initially viewed as resulting from the release of a diffusible endothelial factor, termed EDHF [2], but is now also thought to involve the transmission of endothelial cell hyperpolarization to the underlying vascular smooth muscle cells (VSMCs) *via* myoendothelial gap junctions [3]; the relative importance of each mechanism is thought to vary between different arteries/arterioles and also depends on the degree of pre-existing vascular excitation [4]. In either case, calcium-activated potassium channels K_Ca_2.3 and K_Ca_3.1 (SK_Ca_ and IK_Ca_ respectively) have been shown to play a significant part in the EDH response (EDHR).

Pomposiello et al. [5] suggested that H_2_O_2_ has a role to play in the EDHR, and a little later it was shown that L-NNA- and indomethacin-resistant vasodilatation and hyperpolarization elicited by acetylcholine in mouse small mesenteric arteries was attenuated by catalase and associated with increased endothelial ROS production [6]. Further evidence that H_2_O_2_ acts as an EDHF was also reported for dog coronary resistance arteries [7], [8] and human small mesenteric [9] and coronary arteries [10]. Liu et al. [11] showed that an endothelium-denuded segment of human coronary artery dilated when it was perfused by the effluent from an upstream endothelium-intact artery subjected to increased shear stress, and that this dilatation was attenuated if an H_2_O_2_-scavenging column was interposed between the donor and recipient arteries. The possibility that H_2_O_2_ acts as an EDHF is also consistent with observations by other groups that application of exogenous H_2_O_2_ opens VSMC BK_Ca_ channels [12], hyperpolarizes VSMCs [13] and relaxes a variety of vascular preparations [10], [14], [15], [16]. The evidence for H_2_O_2_ as the primary contributor to the EDHR evoked by bradykinin is also compelling for cerebral arterioles, in which it appears that cyclooxygenase-dependent production of H_2_O_2_ causes vasodilation by acting on BK_Ca_ channels [16], [17]. Most convincingly, Eaton et al. [18] showed that a mouse with knocked-in redox-insensitive protein kinase G were hypertensive and had mesenteric vessels that were less sensitive to applied H_2_O_2_. However, one concern with many of the studies in which the effect of exogenous H_2_O_2_ was examined is that the EC_50_ for H_2_O_2_ – induced vasodilatation of endothelium-denuded arteries was above 10 μM *e.g.* [10], [14], [16] Notably, Liu et al. [11] reported that whereas the vasodilating effluent they collected from endothelium-intact perfused human coronary arteries contained 0.6 μM H_2_O_2_, vasodilatation of endothelium-denuded arteries by exogenous H_2_O_2_ was negligible at a concentration of 10 μM.

Intriguingly, although H_2_O_2_ dilated endothelium-intact human submucosal intestinal microvessels, it caused constriction following endothelial denudation, a finding which led to the suggestion that H_2_O_2_ promotes the release of a separate EDHF instead of itself acting as an EDHF [15]. It was subsequently shown by Griffith and co-workers that the application of 100 μM H_2_O_2_, or the oxidizing agent thimerosal, to rabbit aortic valve endothelial cells (EC) enhanced intracellular Ca^2+^ release evoked by the SERCA inhibitor cyclopiazonic acid (CPA). Moreover, CPA-induced vasodilatation of rabbit ileac arteries was attenuated by application of catalase. They proposed that H_2_O_2_ could potentiate EDH-dependent vasodilatation by increasing IP_3_ - dependent Ca^2+^ release [19]. Soon afterwards this group demonstrated that applying H_2_O_2_ concentrations of 10–30 μM also potentiated the EDH-associated vasodilation caused by acetylcholine in rabbit ileac arteries [20].

These studies suggest strongly that H_2_O_2_ acts to enhance rather than mediate the EDHR, and pose a number of important questions which are currently unanswered. As the ability of H_2_O_2_ to facilitate the EDHR has not been compared to its direct vasodilator effects in any single preparation, it remains unknown which of these effects are likely to be of predominant importance during the EDHR. Also, the pathways leading to H_2_O_2_ production within EC which would facilitate increases in EC [Ca^2+^]_i_ during the EDH response have not been defined. In addition, it is not clear whether physiological stimuli which have been shown to increase ROS production in EC lead to increases in the EDHR as would be predicted by this mechanism.

We have investigated these issues in arterioles in the intact rat cremaster circulation, as this preparation exhibits robust, sustained and consistent dilatations to both carbachol and H_2_O_2_, and permits endothelial Ca^2+^ concentration changes to be measured simultaneously. We characterized the effects of anti-oxidants and blockers of potential ROS-producing pathways on increases in EC [Ca^2+^]_i_ and the associated NO- and PGI_2_-independent vasodilation evoked by the muscarinic receptor agonist carbachol. In addition, we carried out a comparison of the effects of carbachol and exogenously applied H_2_O_2_ in order to determine whether H_2_O_2_ might be acting as an EDHF. We also assessed whether EDH and ROS contribute to the vasodilatation induced by the inflammatory mediator cytokine IL-1β. Our results show that under basal conditions, endogenous ROS production involving CYP92C, phospholipase A_2_ and hydroxyl radical greatly facilitates EC Ca^2+^ release and EDH-associated vasodilation. A brief pre-incubation with IL-1β further enhances the responses to carbachol. Although H_2_O_2_ also dilated these arterioles directly, it only did so at high concentrations, and *via* mechanisms which differ markedly from those evoked by carbachol.

## 2 Methods

This study conforms with the *Guide for the Care and Use of Laboratory Animals* published by the US National Institutes of Health (NIH Publication No. 85–23, revised 1996) and is in accordance with UK Home Office regulations (Animals Scientific Procedures) Act, 1986.

4–6 week old male Wistar rats (Charles River, UK) weighing 80–100 g were killed by exposure to an increasing concentration of CO_2_ followed by cervical dislocation. A midline incision was made along the abdomen and the skin and muscle layer were retracted to expose the underlying organs. The inferior vena cava was tied off above the bifurcation into the common iliac veins and below that an incision was made to allow blood to drain from the lower extremities. A 0.61 mm fine bore polythene cannula was inserted down to the bifurcation into the common iliac arteries *via* the abdominal aorta and secured with thread. The left common iliac and the right femoral and internal iliac arteries were ligated to ensure that perfusion was only to the right external iliac artery supplying the cremaster artery and the cremaster muscle. This was then perfused through the cannula with a stabilizing solution (10 Mg^2+^, 110 NaCl, 8 KCl, 10 HEPES, 1 CaCl^2+^ all mM) containing heparin (30 U/ml) with isoproterenol 10 µM buffered to pH 7.0±0.05 for 10 min to remove all blood.

The cremaster muscle was prepared by a midline incision along the scrotum to expose the right testes. Skin and connective tissue were carefully removed from the underlying cremaster muscle, which was then cut longitudinally along the anterior surface from the apex to the inguinal canal while keeping the vascular supply and drainage at the base intact. The testis was separated from the cremaster muscle and was pulled through the inguinal canal so as not to obstruct the preparation. The muscle was spread out flat onto a clear Sylgard disc using forceps and secured using micro- histology pins, and the cardioplegic solution perfusing the circulation was then replaced for the duration of the experiment with PSS containing 0.1% albumin delivered by a gravity controlled reservoir. The preparation was moved to the stage of a Leitz Intravital microscope equipped with Orthoplan optics, and was superfused with PSS at 37 °C at a flow rate of 2.5 ml/min. PSS contained the Na^+^ channel blocker lidocaine (20 mg l^−1^) to block neural activity and keep the cremaster muscle from contracting.

Following a 30 min equilibration period an appropriate 2nd order arteriole (diameter 50–100 µm) was constricted by superfusion with 30 µM phenylephrine (PE) in combination with 300 µM N(G)-nitro-l-arginine methyl (l-NAME) and 3 µM indomethacin to inhibit NO and PGI_2_ mediated vasodilatation. Constriction was maintained for 20 min to ensure its stability, and the muscarinic receptor agonist carbachol (10 µM) was then added for 2 min to the superfusate to elicit vasodilation. Arterioles were used for experiments only if a vasodilatation of >50% (measured as described below) was observed.

During the experiments PE, l-NAME and indomethacin were present throughout. Drugs used to characterize the EDHR were added to the superfusate for 2–20 min before, and during, the application of carbachol. In experiments examining the effect of IL-1β, the preparation was superfused for 15 min with PSS containing the cytokine at a concentration of 100 pM. IL-1β was then washed out briefly and carbachol was applied.

### 2.1 Diameter measurements and EC [Ca^2+^]_i_ measurements

Arterioles were visualized with a x40 water-immersion objective lens (allowing diameter measurements to be taken to an accuracy of 1 µm) and were perfused for 1 h at room temperature with PSS containing 10 μM Fura-PE 3 AM, 1% DMSO, 0.02% pluronic acid and 1% albumin in order to selectively load the endothelium with furaPE3. The preparation was then perfused with PSS and the superfusate temperature was raised to 37 °C. Following a 15 min equilibration and washout period, arterioles were illuminated alternately at 360 and 380 nm at 1 Hz using a filter wheel (Cairn Research Ltd, UK) and images were recorded at >530 nm using an Intensified ISIS camera (Photonic Sciences, UK). Images were analyzed using ImageHopper (Samsara Research: Dorking, Surrey, UK) software. Diameter was measured using the inner margin of the 380 nm signal. A semi-quantitative calculation of the endothelial Ca^2+^ concentration (hereafter termed EC [Ca^2+^]_i_) was made by subtracting the average background (adjacent to the arterioles) signal from the image, normalizing the signals from the vessel during the 360 and 380 nm excitation intervals, and calculating the 360/380 ratio at each pixel. PE had no effect on the 360/380 ratio, which indicates that FuraPE3 was confined to the endothelium. Furthermore, carbachol and TRAM-34 co-applied with apamin produced changes in the 360/380 ratio that would be expected from selective FuraPE3 endothelium loading.

### 2.2 Drugs and solutions

PSS contained (in mM) 124 NaCl, 5 KCl, 22 NaHCO_3_, 5 glucose, 2 MgSO_4_, 0.125 NaH_2_PO_4_, 2 CaCl_2_, and was gassed with 5% CO_2_ - balance air to maintain the pH at 7.40. St. Thomas’ cardioplegic (stabilizing) solution contained (in mM) 110 NaCl, 7.9 KCl 34 MgCl_2_, 1 CaCl_2_, 11 HEPES, 0.01 isoprenaline and 0.01 ascorbic acid as an antioxidant. Drugs were made up freshly each day or made from stock solutions stored at −20 °C with final concentrations made up in PSS. All drugs were purchased from Sigma Aldrich UK, other than: TRPM2 antibody (Cambridge Bioscience), arachidonyl trifluoromethyl ketone (AACOCF_3_: Merck Chemicals), heparin (AAH Hospital Services), TRAM-34 (Tocris Bioscience) and lidocaine (Braun Medical). CP94 (1,2-diethyl-3-hyroxypyridin-4-one) and CP85 (Na-1-ethylsulfoxide-2-methyl-3-hyroxypyridin-4-one) were kind gifts from Professor R.C. Hider, King's College London.

### 2.3 Presentation of data and statistical analysis

Endothelial cell [Ca^2+^]_i_ is represented as the peak % increase in the Fura-PE3 360/380 ratio from the baseline. Vasodilatation is represented as the maximum % reversal of PE-induced constriction. Carbachol responses were recorded under control conditions and then again following an intervention (*e.g.* application of an inhibitor), so that each arteriole acted as its own control and results could be analyzed using a two-tailed paired *t*-test. It was possible to do so because the responses evoked by carbachol were stable when repeated (rise in Fura-PE3 360/380 ratio and vasodilation of 20.5±2.4% and 75.3±3.5%, respectively, during first application of carbachol; rise in Fura-PE3 360/380 ratio and vasodilation of 22.1±2.6% and 72.5±3.2%, respectively during second response to carbachol applied 15 min later, n=21, ns). Bar charts represent mean±S.E.M values.

## 3 Results

### 3.1 Role of ROS in EDH-associated carbachol-induced vasodilation of rat cremaster arterioles

**Fig. 1:**
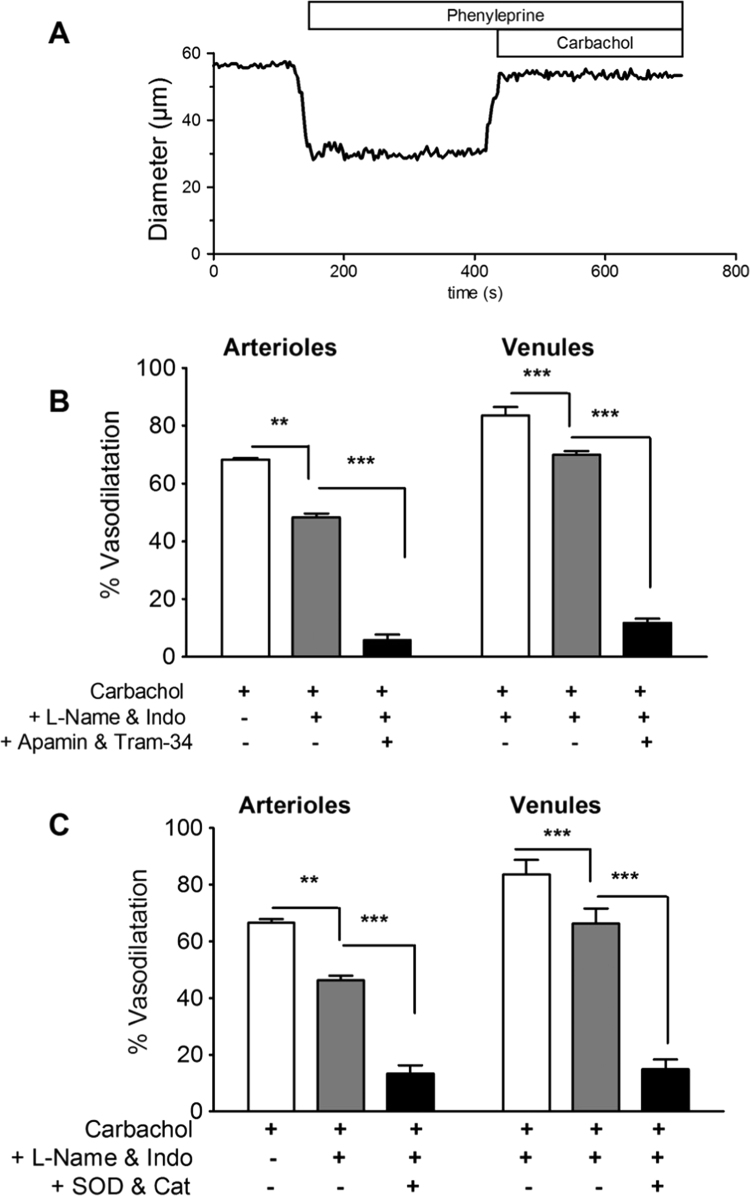
Properties of the carbachol-induced EDHR in rat cremaster microvasculature. (A) Representative experiment illustrating the constricting effect of applying phenylephrine (30 µM) and the subsequent sustained dilating action of carbachol (10 µM). (B) Paired experiments in which the dilatation induced by carbachol was reduced by co-applying l-NAME (300 µM) with indomethacin (3 µM), and subsequent apamin (0.5 µM) with Tram-34 (10 µM) or (C) a ROS scavenging mixture of superoxide dismutase and catalase (100 U/ml each). Analysis of variance followed by Tukey's multiple comparison test ***p<0.001, **p<0.01.

PE (30 µM) application to cremaster resulted in a sustained constriction (36±3%, n=5), and subsequent carbachol (10 µM) application resulted in a well-maintained vasodilatation, amounting to a 72.6±2.4% reversal of the constriction (n=55). Fig. 1A shows an example of this, and Figs. 1B and 1C illustrate the results of 4 paired experiments in which the vasodilatation of arterioles and their accompanying venules was measured. The vasodilatation was reduced somewhat in the presence of l-NAME (300 µM) and indomethacin (3 µM) (Fig. 1B). Interestingly, this was less in the venules than the arterioles (arteriolar vasodilatation reduction 30±1.3%, venular reduction 19±1.7%; p<0.001 *t*-test, 8 paired vessels). Addition of apamin (0.5 µM) with Tram-34 (10 µM) to block endothelial SK_Ca_ (K_Ca_2.3) and IK_Ca_ (K_Ca_3.2) channels respectively, resulted in a much greater reduction: a further 62±3.3% in arterioles and 70±1.3% in venules (p<0.05 *t*-test, 4 paired vessels). When a free radical scavenging mixture of superoxide dismutase (SOD) and catalase (S&C; both 100 U/ml) was used instead of Tram-34 and apamin (Fig. 1C) there was a similar reduction in carbachol-induced vasodilatation (arterioles 49±5.3% and venules 62±5.2% 4 paired vessels, ns).

The carbachol-induced arteriolar vasodilatation was accompanied by a sustained increase in EC [Ca^2+^]_i_ which was not affected by the presence of l-NAME and indomethacin (24.5±5.4 *vs* 24.1±4.6% increase, n=4, ns). All experiments described hereafter were carried out in the presence of l-NAME and indomethacin in order to allow the EDH-associated vasodilatation to be recorded in isolation.

**Fig. 2:**
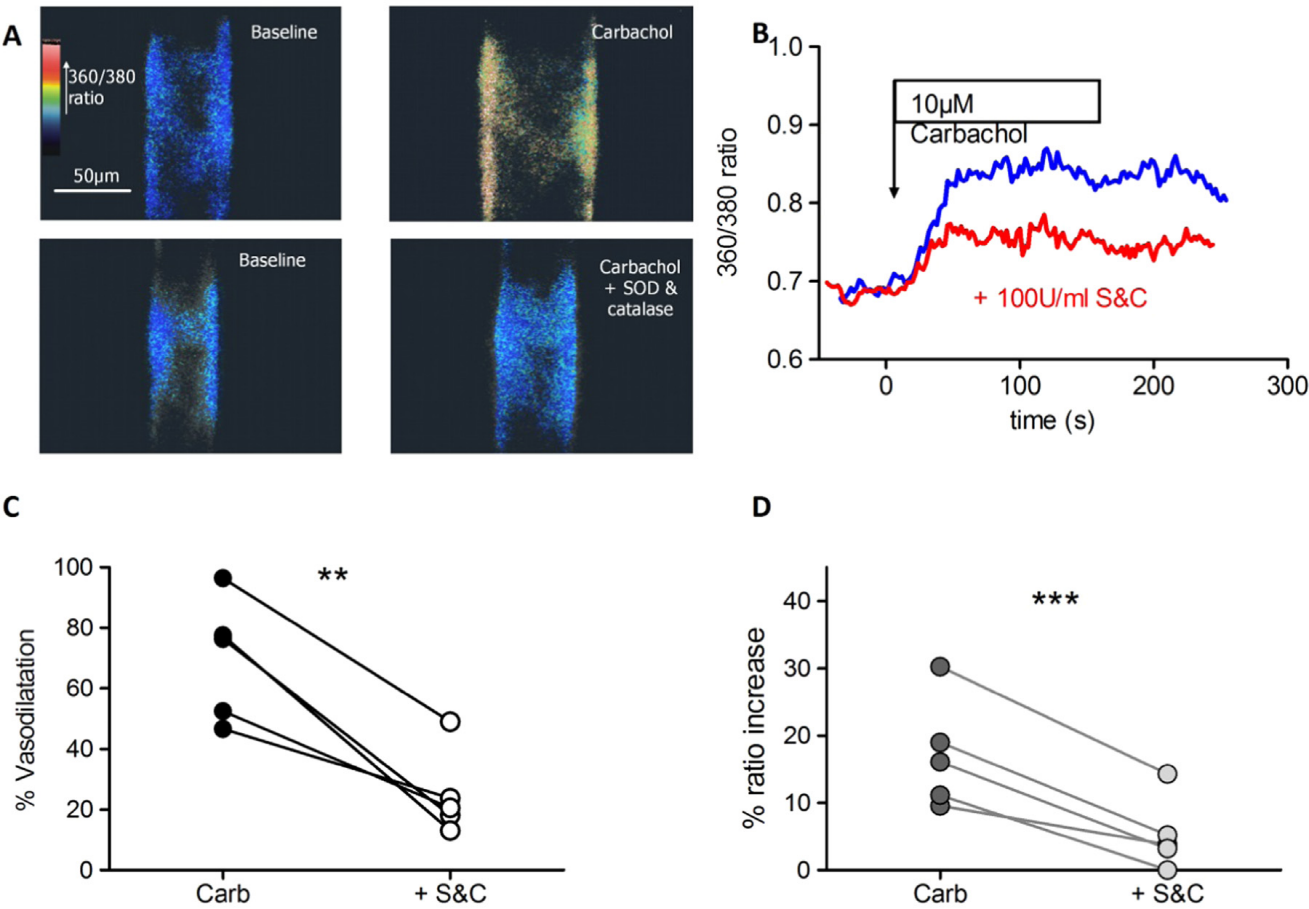
Carbachol-induced EDHF response is ROS dependent. (A) Example of the effect of S&C (both 100 U/ml) on the carbachol-mediated rise in EC [Ca^2+^]_i_ and vasodilatation in an arteriole, and (B) the time course of the carbachol-induced rise in the 360/380 nm ratio and its attenuation by S&C. (C) S&C reduces the vasodilatation and (D) the endothelial [Ca^2+^]_i_ responses to carbachol (n=6) paired *t*-test, ***p<0.001, **p<0.01).

As shown in Fig. 2, both the rise in EC [Ca^2+^]_i_ and the vasodilatation induced by carbachol were greatly diminished in the presence of S&C. Fig. 2A illustrates images of the 360/380 fluorescence emission ratio in a Fura PE3 loaded cremaster arteriole in the presence of PE (upper left), and after 10 μM carbachol was applied (upper right). The arteriole was subsequently washed with PSS, superfused with PE in the presence of S&C (lower left), and again exposed to carbachol (lower right). The effect of S&C on the carbachol-induced increase in EC [Ca^2+^]_i_ was sustained, as shown in Fig. 2B. Fig. 2C shows that in six paired vessels that S&C application reduced the carbachol-induced vasodilatation (left) and increase in EC [Ca^2+^]_i_ (right) on each occasion.

**Fig. 3:**
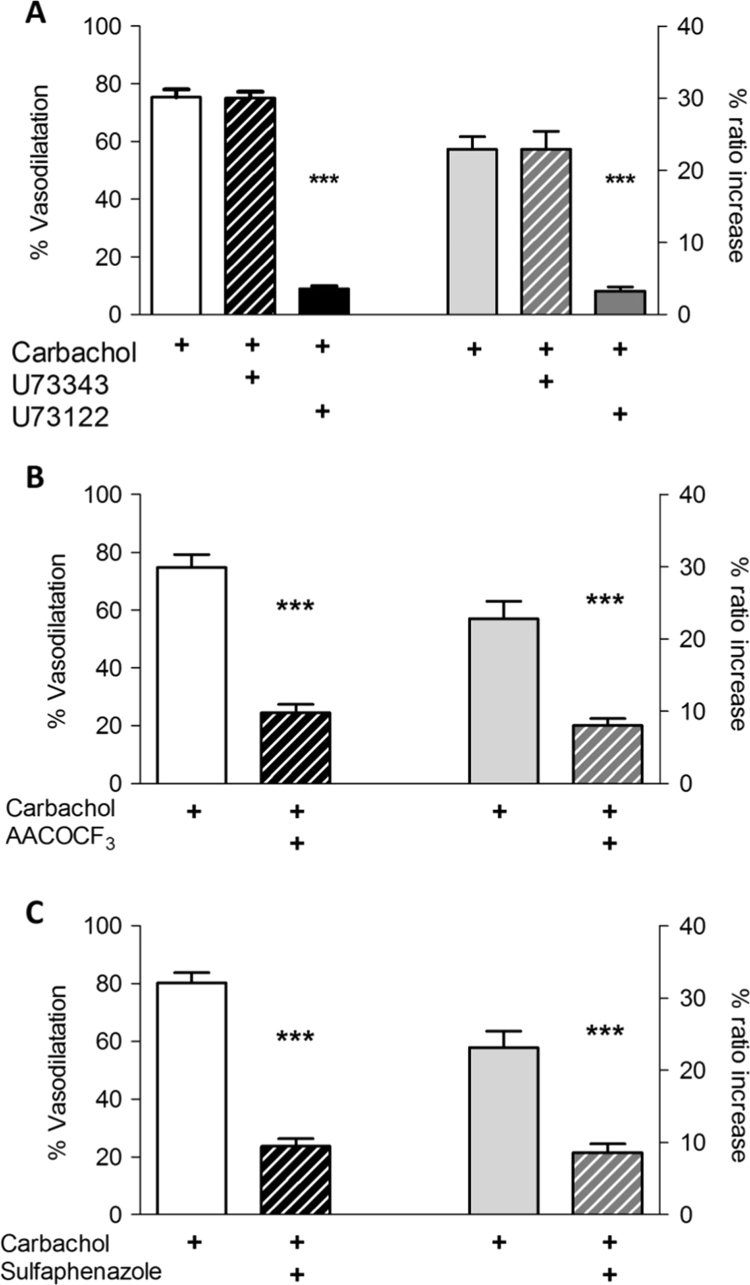
Paths by which carbachol induces EDHF response. (A) The PLC inhibitor U73122, but not the structurally similar U73343 (both 3 µM), very much reduced the [Ca^2+^]_i_ (from 22.3±3.2%) and the vasodilatation (from 75.3±2.7 to 10.4±1.9%) responses to carbachol (10 µM). (B) The PLA_2_ inhibitor AACOCF_3_ (3 µM) and (C) the cytochrome P450 2C9 inhibitor sulfaphenazole (10 µM) also reduced the [Ca^2+^]_i_ and vasodilatation responses. 5 vessels in 5 animals for each data set; paired *t*-tests *** p<0.001. The reduction with U73122 was greater than with either AACOCF_3_ or sulfaphenazole (see text).

Carbachol stimulates muscarinic M_1_ and M_2_ receptors which will result in activating phospholipase C and phospholipase A_2_ respectively. Inhibiting PLC with 3 μM U73122 resulted in much reduced vasodilatation and Ca^2+^ responses (to 11.6±1.1% and 13.8±1.8%, respectively), whereas the inactive U73122 analogue U73343 (3 μM) had no effect on either response (Fig. 3A). This suggests that store release of Ca^2+^ is a key requirement for the EDH response. Similarly PLA_2_ inhibition by 3 μM AACOCF_3_ also reduced the vasodilatation and Ca^2+^ responses (Fig. 3B), but to a lesser extent. The arachidonic acid released by PLA_2_ can interact with a number of enzymes to generate reactive oxygen species (ROS), notably cyclooxygenases, lipoxygenases, xanthine oxidase, and cytochrome P450 isoforms. We used a number of inhibitors to investigate possible sources, namely allopurinol (xanthine oxidase), nordihydroguaiaretic acid (lipoxygenase), and sulfaphenazole (cytochrome P450), while indomethacin was always present to block cyclooxygenase. Of these only sulfaphenazole, a selective cytochrome P450 2C9 inhibitor [21] reduced the responses to carbachol (Fig. 3C). The less specific inhibitors 2-(2-propynyloxy)-benzenehexanoic acid (PPOH) and 17-octadecynoc acid (ODYA) were equally effective (Supplementary Fig. 1). These effects were not due to a reduction in the production of EETs, since neither the EETs antagonist 4, 15-epoxyeicosa-5(Z)-enoic acid (EEZE) nor the soluble epoxide hydrolase (sEH) blocker 12-(3-adamantan-1-yl-ureido)dodecanoic acid (AUDA) had any effect on the carbachol responses (Supplementary Fig. 2B). It is interesting to note that the reduction in vasodilatation and the Ca^2+^ response with AACOCF_3_ and sulfaphenazole was significantly less than with PLC inhibition (AACOCF_3_ vasodilatation 25.6±6.9% and EC [Ca^2+^]_i_ 24.9±4.1%; sulfaphenazole vasodilatation 30.1±4.4% and EC [Ca^2+^]_i_ 37.2±3.7%) (Anova: EC [Ca^2+^]_i_ P<0.005; vasodilatation P<0.05).

### 3.2 H_2_O_2_ assists release of Ca^2+^ from intracellular stores

**Fig. 4:**
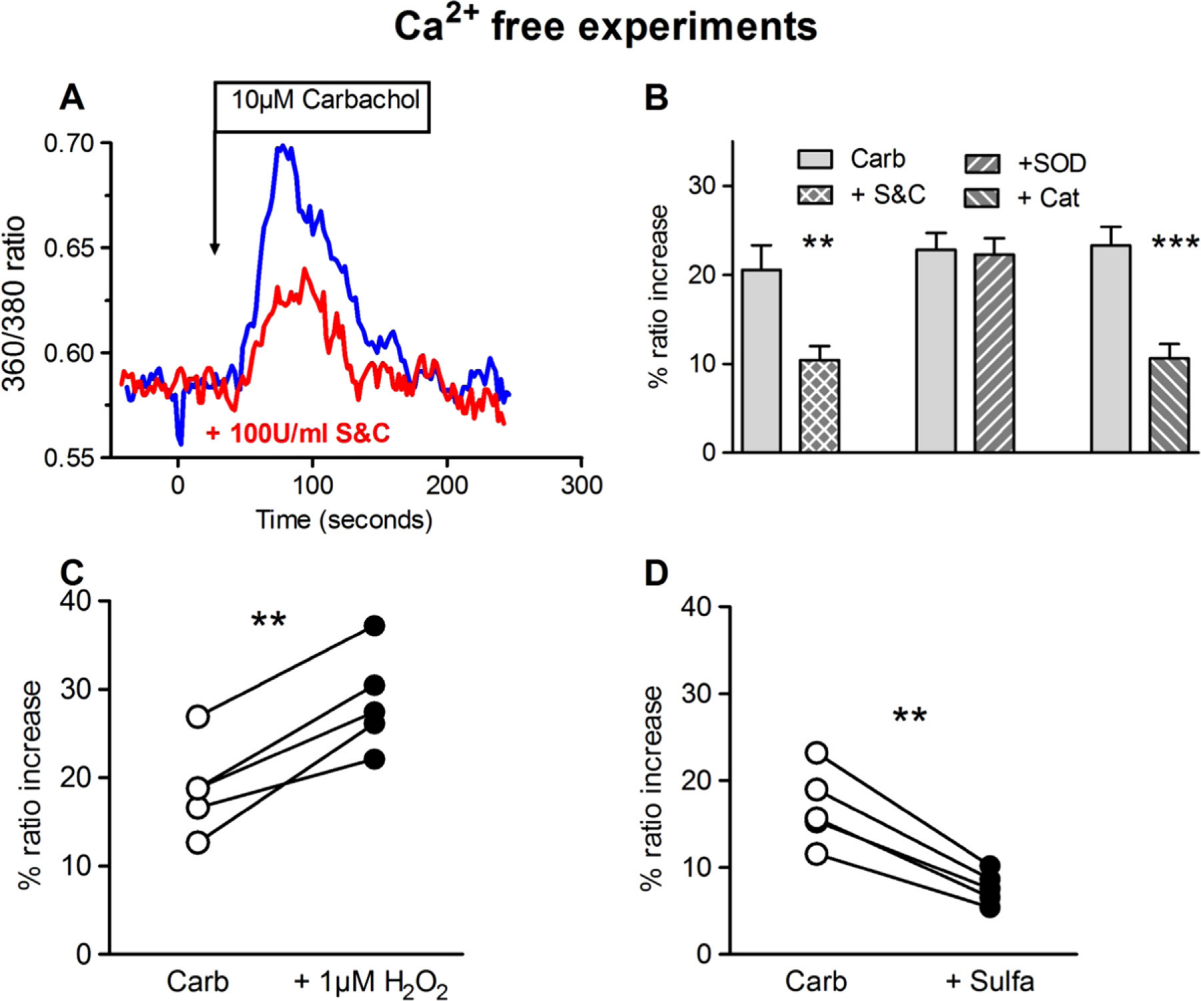
Role of ROS on endothelial Ca^2+^ release using Ca^2+^-free Krebs buffer. (A) Example of the effect of S&C (both 100 U/ml) on the carbachol-induced rise in EC [Ca^2+^]_i_. (B) Average effect of SOD and catalase applied in combination or independently on the peak carbachol-induced transient rise in EC [Ca^2+^]_i_. (C) Effect of 1 µM H_2_O_2_ and (D) of sulfaphenazole (10 µM) on the carbachol induced increase in EC [Ca2^+^]_i_. (n=5 for B-D, paired *t*-test **p<0.01, ***p<0.001).

Carbachol-induced release of intracellular Ca^2+^ by the endothelium was recorded in arterioles perfused and superfused with Ca^2+^ free PSS containing 0.2 mM EGTA in order to prevent Ca^2+^ influx. Under these conditions carbachol caused a transient increase in EC [Ca^2+^]_i_, which was significantly attenuated by S&C (Fig. 4A and B). Further experiments revealed that the increase in EC [Ca^2+^]_i_ was similarly reduced by catalase alone, but was unaffected by SOD (Fig. 4B). Since reducing H_2_O_2_ levels suppressed the carbachol-induced increase in EC [Ca^2+^]_i_ and the associated vasodilation, it might be predicted that increasing H_2_O_2_ levels should correspondingly facilitate the response to carbachol. As depicted in Fig. 4C, application of 1 μM exogenous H_2_O_2_ significantly increased the transient carbachol-induced rise in EC [Ca^2+^]_i_ in Ca^2+^-free PSS. The role of cytochrome P450 2C9 in assisting Ca^2+^ release was confirmed by the inhibitory action of sulfaphenazole (Fig. 4D) on this response.

**Fig. 5:**
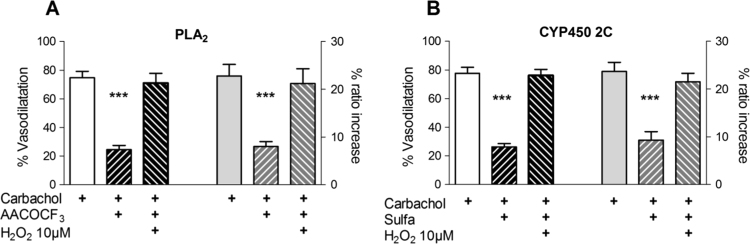
Reversal of the effects of ROS-generating enzyme inhibition by H_2_O_2_. 10 μM H_2_O_2_, which alone had no effect on vessel diameter, was applied to arterioles where (A) AACOCF3 (3 µM) or (B) sulphaphenazole (10 µM) had reduced the carbachol induced vasodilatation (left) and rise in EC [Ca^2+^]_i_ (right). In both cases, H_2_O_2_ fully reversed the effects of the inhibitors. (5 vessels from 5 animals; paired *t*-test *** p<0.001).

**Fig. 6:**
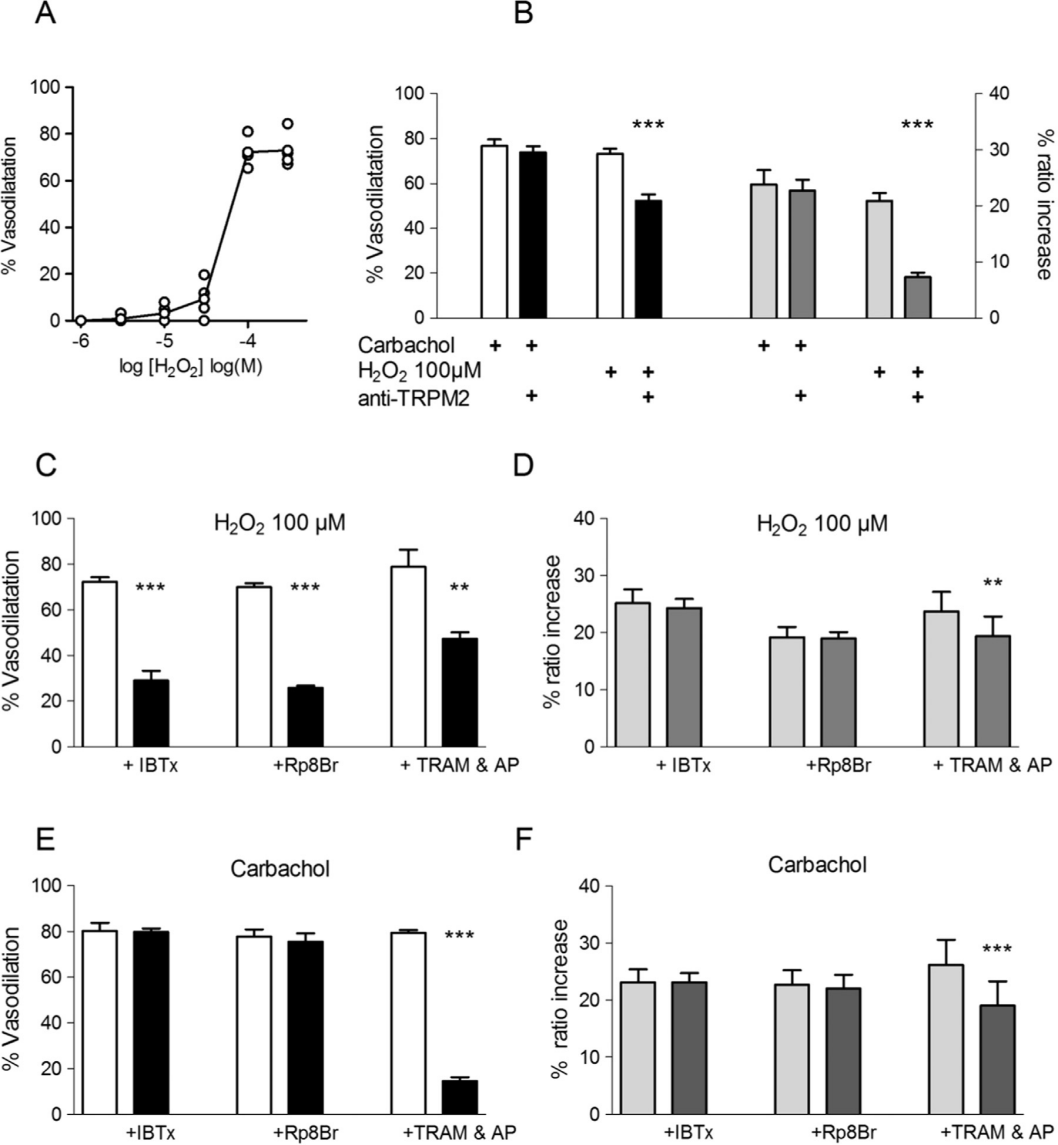
Effects of exogenous hydrogen peroxide. (A) Concentration dependency of H_2_O_2_ induced vasodilation with 300 µM l-NAME and 10 µM indomethacin present (n=4). (B) Pre-incubation with TRPM2 antibody (5 µg ml^−1^) reduced the vasodilatation and Ca^2+^ responses to exogenous H_2_O_2_, but not to carbachol. (C) H_2_O_2_ caused vasodilatation that was inhibited by Rp-8bromo cGMP (100 µM), IBTx (100 nM), but these had no effect on EC [Ca^2+^]_i_. (D) The combination of TRAM34 (10 µM) and apamin (0.5 µM) reduced the vasodilatation and Ca^2+^ responses. In contrast, neither Rp-8bromo cGMP nor IBTx had any influence on the carbachol, responses (E & F). n=5–9 vessels, each from separate animals.

The results depicted in Fig. 3, Fig. 4 are consistent with the concept that arachidonic acid released by PLA_2_ is metabolized by CYP2C9 to produce ROS, which then potentiate carbachol-induced Ca^2+^ release. To further test this idea we added H_2_O_2_ (10 µM, a concentration that produced no vasodilatation on its own (see next section and Fig. 6A) to preparations where either PLA_2_ or CYP2C9 were blocked with AACOOCF_3_ or sulphaphenazole, respectively. In both cases H_2_O_2_ fully restored vasodilatation and Ca^2+^ responses (see Fig. 5A & B).

### 3.3 Properties of the response to H_2_O_2_ applied exogenously on its own

Application of H_2_O_2_ to PE-preconstricted arterioles caused a steeply concentration-dependent vasodilatation, with an apparent threshold at ~30 μM and maximum at 100 μM H_2_O_2_ (Fig. 6A). When evaluated in a larger number of arteries, vasodilatation at 100 μM H_2_O_2_ was similar to but slightly smaller than that induced by 10 μM carbachol (73.7±1.4% *vs* 80.8±1.4%, n=22, p<0.05), and was accompanied by a rise in EC [Ca^2+^]_i_ which was also somewhat smaller than that caused by carbachol (22.6±1.4% *vs* 26.0±1.4%, n=22, p <0.05).

The possibility that the exogenously applied H_2_O_2_ resulted in opening the TRPM2 channel was tested by using a blocking antibody that had been shown to block H_2_O_2_-induced Ca^2+^ influx into cultured endothelial cells [22]. It is interesting to note that the EC_50_ for activation of this channel by H_2_O_2_ (~60 µM), is consistent with the dose-response relationship we found (Fig. 6A). Application of the TRPM2 antibody reduced both the H_2_O_2_-induced rise in EC [Ca^2+^]_i_ and vasodilatation, but left these responses to carbachol unaffected (Fig. 6B).

Although both 100 μM H_2_O_2_ and carbachol caused roughly equivalent effects on EC [Ca^2+^]_i_ and vascular diameter, they apparently did so through different mechanisms. As shown in Figs. 6C and 6D, the vasodilatation elicited by H_2_O_2_ was strongly suppressed by the protein kinase G blocker Rp-8-Br-cyclic GMP (100 μM) and the BK_Ca_ channel inhibitor IBTx, (100 nM), and was to a smaller extent also sensitive to the combination of apamin (500 nM) and TRAM-34 (10 µM). Conversely, Rp 8Br-cyclic GMP and IBTx had no effect on the H_2_O_2_-induced rise in EC [Ca^2+^]_i_, which was strongly supressed by the TRPM2 antibody and TRAM-34+apamin. Inhibiting PKG and the BK_Ca_ channel, however, had no effect on the carbachol-mediated responses (Figs. 6E and 6F). TRAM-34 and apamin reduced vasodilatation mediated by H_2_O_2_ (−37±2.8%), to an extent which was which was significantly less than that observed for the response to carbachol (−82±2.8%; P<0.001).

In additional experiments we found that CP94, which inhibited carbachol induced vasodilation (see below), had no affect on dilatation to H_2_O_2_ (78.0±2.5% in H_2_O_2_ *vs* 77.0±2.1% in H_2_O_2_ with CP94, n=5, ns). H_2_O_2_-induced vasodilation was also unaffected by AACOCF_3_ (69.8±1.8% in H_2_O_2_ *vs* 68.7±1.8% in H_2_O_2_+AACOCF_3_, n=5, ns).

### 3.4 The nature of the ROS involved in carbachol-mediated effects

**Fig. 7:**
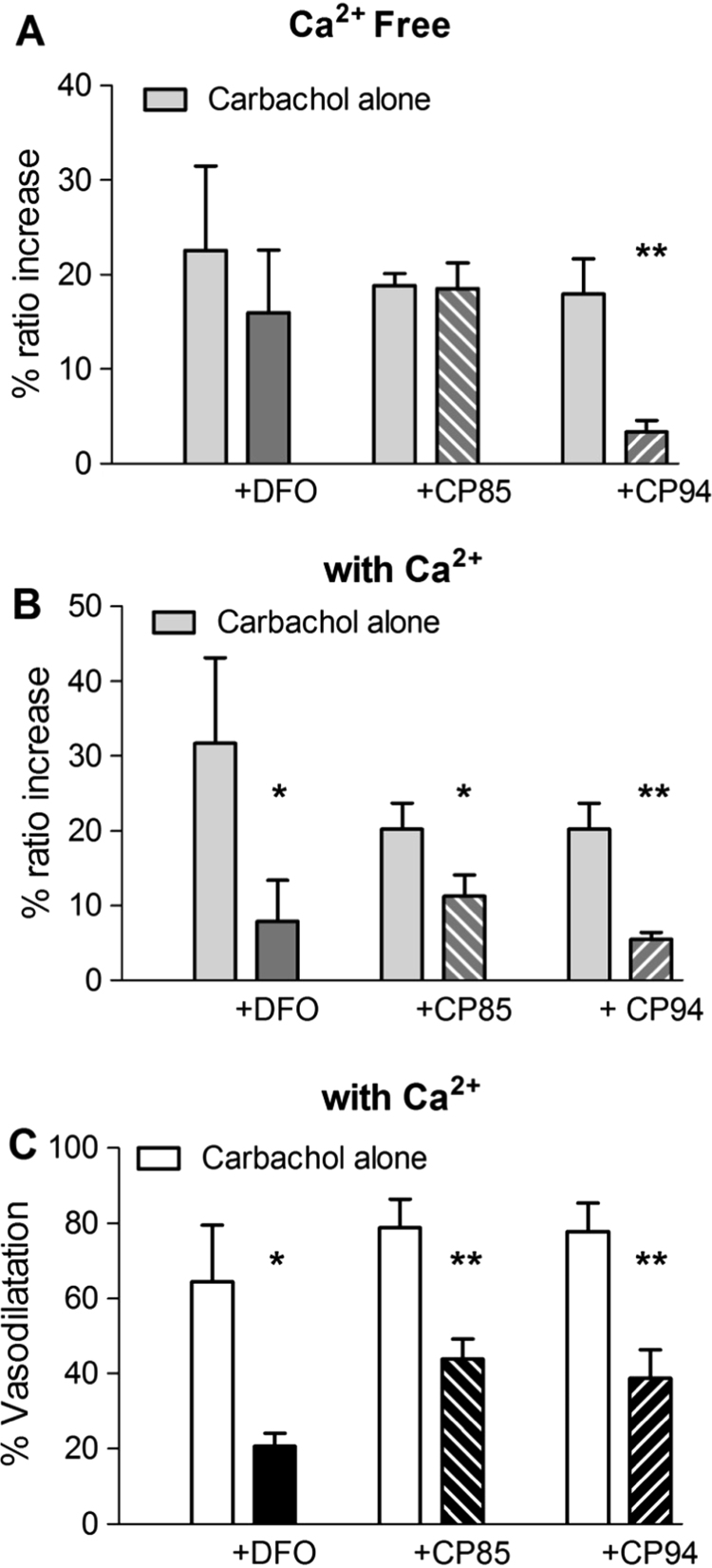
Effects of iron chelation on carbachol responses. Effects of the cell permeable CP94 and the cell impermeable desferrioxamine, CP85 (each 100 µM) iron chelators on carbachol responses. (A) In Ca^2+^-free PSS the induced rise in the EC [Ca^2+^]_i_ is reduced only with CP94. (B) When Ca^2+^ was included in the medium all the chelators reduced the [Ca^2+^]_i_ and the (C) vasodilatation responses. (5 vessels from 5 animals; paired t test *p<0.05, **p<0.01).

Desferrioxamine and the hydroxypyridinones CP94 and CP85 are selective iron chelators, but differ in that CP94 is cell permeable while the other two are not [49]. As shown in Fig. 7A, CP94 (100 μM) significantly reduced the transient carbachol-induced rise in EC [Ca^2+^]_i_ in Ca^2+^ free PSS, while neither desferrioxamine nor CP85 (100 μM) had any effect on this response. This supports the view that ∙OH, rather than H_2_O_2_ itself, is the radical responsible for Ca^2+^ release from stores.

In the presence of extracellular Ca^2+^, all three iron chelators inhibited the carbachol-induced rise in endothelial [Ca^2+^]_i_ (Fig. 7B) and reduced the accompanying vasodilation (Fig. 7C). Notably, none of these substances had any effect on the PE induced contraction (not shown).

### 3.5 IL-1β causes ROS-dependent vasodilatation and elevation of EC [Ca^2+^]_i_

**Fig. 8:**
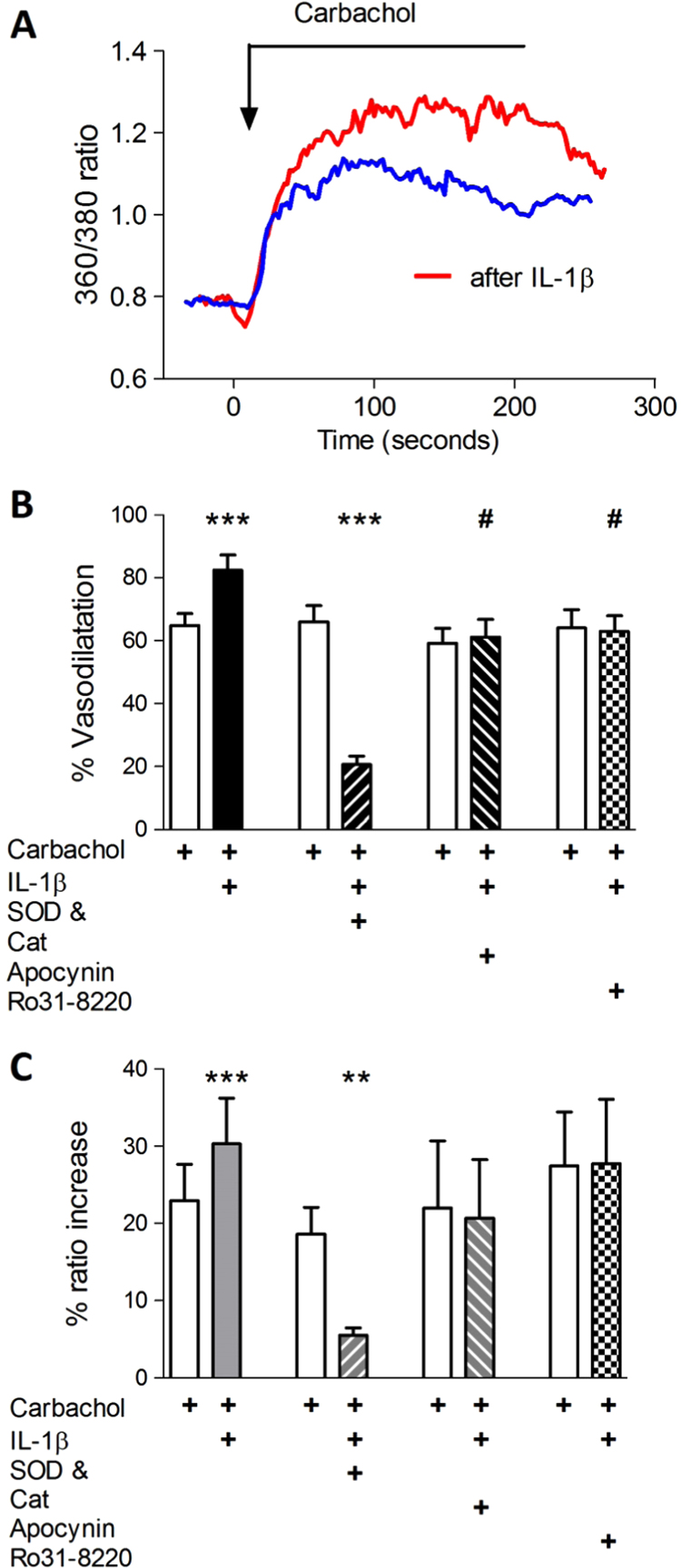
Evidence that IL-1β increases the response to carbachol *via* NADPH oxidase. IL-1β (30 pM) applied for 15 min. resulted in increased (A) vasodilatation and (B) EC [Ca^2+^]_I_ responses. Co-application with either apocynin (1 µM) or Ro31-8220 (3 µM) blocked the increases, while a radical scavenging mixture of SOD and catalase (100 U/ml each) reduced the total responses. (5 vessels from 5 animals; paired *t*-tests **p<0.01; ***p<0.001; IL-1β *vs* IL-1β +apocynin # unpaired ‘*t*’ test p<0.05.

We have previously shown that a brief application of a low concentration of interleukin-1β (30 pM for 10 min) results in endothelial ROS generation *via* NADPH oxidase [23], and that this is prevented by co-application with a low concentration of apocynin (1 µM; [24]). As shown in Fig. 8, a 15 min pre-incubation of the preparation with IL-1β (30 pM), followed by a subsequent brief wash to remove the cytokine, resulted in increased EC [Ca^2+^]_i_ and vasodilatation responses that were much reduced by scavenging ROS. Furthermore, this potentiating effect was abolished when apocynin (1 μM) was co-applied and washed off with IL-1β. NADPH oxidase assembly requires phosphorylation of p47^*phox*^ by protein kinase C [25], and when the broad spectrum PKC inhibitor Ro31-8220 (3 μM) was applied with IL-1β, the potentiation was also abolished. Neither apocynin nor Ro31-8220 affected the response to carbachol in the absence of IL-1β (Supplementary Fig. 3).

## 4 Discussion

The key finding of this study is that whilst the EDHR triggered by carbachol is largely ROS-dependent, it does not involve an effect on VSMCs of H_2_O_2_ released by the endothelium. Instead, the results demonstrate that ROS generated by endothelial cytochrome P450 2C9 make a crucial contribution to the EDHR triggered by muscarinic receptor stimulation in rat cremaster arterioles. They do this by acting on the endothelium to potentiate Ca^2+^ release from intracellular stores and enhancing Ca^2+^ entry across the plasma membrane rather than by acting directly as an EDHF. There have been previous indications that this is an important mechanism: thus catalase suppresses cyclopiazonic acid (CPA) induced Ca^2+^ release in rabbit aortic valve endothelium, as well as EDH-dependent, acetylcholine-induced vasodilatation of rabbit iliac arteries. Furthermore, raising the H_2_O_2_ concentration facilitates acetylcholine and CPA induced vasodilatation [19], [20]. CPA induced Ca^2+^ release was also shown to be potentiated by the IP_3_R sensitising agent thimerosal, which implies that H_2_O_2_ may be exerting a similar sensitising effect, as described for the IP_3_R in human aortic endothelial cells [26]. Based on these and related findings, the late Tudor Griffith and colleagues proposed that H_2_O_2_ was acting to promote the EDH response by facilitating IP_3_R-mediated endothelial Ca^2+^ release and thereby raising [Ca^2+^]_I_ [19].

### 4.1 Phospholipase C, Phospholipase A_2_ and CYP450 2C9

Carbachol activates both M_1_ and M_2_ receptors, which are linked to phospholipase C and phospholipase A_2_ respectively. The present findings indicate that both are required for the full EDHR. We showed that blocking PLC with U73122 inhibits increases in both endothelial [Ca^2+^]_i_ and vasodilatation (see Fig. 3A), which is consistent with the finding that many agonists evoke vasodilatation and [Ca^2+^]_i_ increases that are dependent on endothelial PLC activation [27]. Our results are consistent with those of Fukao et al. who showed that PLC is crucial for the EDHR in rat mesenteric arteries, implying that IP_3_R-mediated Ca^2+^ release is pivotal for both events [28].

We have also shown that a pathway involving both cPLA_2_ and CYP2C9 is the source for ROS in the carbachol-induced EDHR in these arterioles. Our results (Fig. 3B), demonstrate that the cPLA_2_ inhibitor AACOCF_3_ suppressed the carbachol-induced EDHR. A role for cPLA_2_ in EDH has previously been demonstrated in some [29], [30], [31] but not all arteries [32]. Although this could reflect a role for EETs generated as CYP450 metabolises arachidonic acid produced by cPLA_2_, it may also depend upon ROS produced the same pathway. This possibility was investigated in porcine coronary arteries, but it was found that this did not apply to the EDHR triggered by bradykinin [5]. In contrast, increases in endothelial permeability evoked by bradykinin in cerebral venular capillaries were found to depend on ROS generated when arachidonic acid produced by cPLA_2_ is metabolised by cyclooxygenase and lipoxygenase [33].

CYP450 has been thought to be involved in the EDH response by virtue of its production of EETs, which were initially proposed to act *via* opening BK_Ca_ channels in vascular smooth muscle cells *e.g.* [34], [35]. More recently however, EETs generated by CYP450 2C9 were shown to act within endothelial cells by increasing [Ca^2+^]_i_ by recruiting TRP channels to the plasmalemma [21], [36].

On the other hand, CYP450 enzymes are also a source of ROS [37], and furthermore, basal ROS production by endothelial cells is dependent on the CYP450 2C9 isoform, as shown by using antisense oligonucleotides [35] and sulfaphenazole [37], both of which suppress EDH. Nevertheless, the possibility that CYP450 2C9 might promote EDH *via* ROS generation [38], as indicated by the results depicted in Fig. 3C, seems not to have been previously considered. Sulfaphenazole strongly suppressed carbachol-induced Ca^2+^ release, as well as the rise in EC [Ca^2+^]_i_ and the associated vasodilatation in normal Ca^2+^ solution, and neither the EETs antagonist EEZE nor AUDA had any effect on the carbachol responses (Supplementary Fig. 2B). Moreover, although in accordance with previous reports *e.g.* [39] we observed that BK_Ca_ channel blockade with IBTx inhibited the vasodilatation caused by application of exogenous 11, 12 EET, IBTx had no effect on the carbachol-induced vasodilatation (Supplementary Fig. 2A).

### 4.2 Exogenously applied H_2_O_2_

Whereas the experiments with anti-oxidants and blockers of endothelial ROS-producing pathways indicate that H_2_O_2_ acts by potentiating the carbachol-induced EC [Ca^2+^] increase, they do not exclude an additional direct effect of H_2_O_2_ on arteriolar vascular smooth muscle during EDH. We therefore carried out a systematic comparison of the properties of the vasodilator responses to H_2_O_2_ and carbachol-induced EDH. The dose response relationship between H_2_O_2_ and vasodilatation (Fig. 6A) is consistent with most others that do not show significant vasodilatation below 50 µM *e.g.* [6], [11]. The vasodilatation caused by H_2_O_2_ was strongly attenuated by Rp-8Br-cGMP and IBTx (see Fig. 6C). Neither of these blockers had any effect on the H_2_O_2_ induced rise in endothelial [Ca^2+^]_i_ (see Fig. 6D), consistent with the concept that H_2_O_2_ evokes vasodilatation principally via vascular smooth muscle cells by causing direct oxidation-induced stimulation of G kinase [18], this leading to cGMP-mediated opening of BK_Ca_ channels [40]. In contrast, responses to carbachol were insensitive to these agents, implying that any release of H_2_O_2_ on its own by the endothelium was too small to activate this pathway. It appears, however, that the rise in endothelial [Ca^2+^]_i_ triggered by 100 μM H_2_O_2_ also makes a significant contribution to the vasodilatation, since the combination of TRAM-34 and apamin reduced vasodilation to H_2_O_2_. Moreover, a TRPM2 antibody previously shown to block H_2_O_2_-induced rises in [Ca^2+^]_i_ associated with activation of these channels in cultured EC greatly suppressed the H_2_O_2_-induced rise in EC [Ca^2+^]_i_ and reduced the accompanying vasodilatation [22]. These results suggest that the endothelium-dependent component of the vasodilatation to 100 μM H_2_O_2_ in these arterioles was largely caused by the opening of EC TRPM2 channels, leading to a rise in EC [Ca^2+^]_i_ and a consequent EDHR. This would be expected to open endothelial K_Ca_ channels, accounting for the small inhibition of the H_2_O_2_ induced rise in endothelial [Ca^2+^]_i_ caused by TRAM-34+apamin (Figs. 6D, 6F). The observation that the TRPM2 antibody had no effect on either the rise in endothelial [Ca^2+^]_i_ or the vasodilatation induced by carbachol (Fig. 5B) further highlights the difference between the pathways by which carbachol and H_2_O_2_ regulate endothelial [Ca^2+^]_i_.

It has been argued that a relatively high exogenously applied H_2_O_2_ concentration does not accurately reflect the true intracellular H_2_O_2_ due to the effects of peroxidases and a hypothesized barrier to H_2_O_2_ entry across the plasmalemma [41]. On the other hand, H_2_O_2_ has been shown to have a high permeability coefficient (1.6×10^−3^ cm s^−1^) across the EC membrane [42]. Furthermore, we found that the application of just 1 µM H_2_O_2_ resulted in a significant increase in carbachol-induced Ca^2+^ release from intracellular stores (see Fig. 4C). Notably, studies have shown that H_2_O_2_ at concentrations ≤100 μM does not itself cause EC Ca^2+^ release in the absence of a co-stimulus. Hu et al. [26] measured the content of the Ca^2+^ stores in permeabilized cultured human aortic endothelial cells (HAEC) using Mag-indo, and found that this was not affected by 100 μM H_2_O_2_, although H_2_O_2_ at concentrations above 3 μM sensitized these cells to IP_3_-induced Ca^2+^ release and 10 μM H_2_O_2_ promoted histamine-mediated Ca^2+^ release in intact HAEC [43]. Similarly it was found that 100 µM H_2_O_2_ had no effect on EC Ca^2+^ store content, measured using Mag fluo-4, in intact rabbit aortic valve endothelium [19].

Similarly, the effects of inhibiting either PLA_2_ with AACOCF_3_, or CYP450 2C9 with sulfaphenazole were completely reversed by adding H_2_O_2_ (10 μM), a concentration which on its own had no effect (see Figs. 5A and 5B). Thus, it is clear that the suppression of the carbachol-induced EDHR by sulfaphenazole is best explained by a decrease in endothelial H_2_O_2_ rather than the inhibition of EET release it causes. This effect would explain the finding that the EDH-dependent vasodilatation to acetylcholine in guinea-pig coronary arteries was antagonised by CYP450 blockers but not IBTx [39], as well as the observation that sulfaphenazole-induced attenuation of the EDHR triggered by acetylcholine and bradykinin in the human forearm microcirculation was prevented by co-administration of the anti-oxidant ascorbate [44].

These findings indicate that ROS produced when CYP 450 2C9 metabolises arachidonic acid generated by cPLA_2_ are responsible for an increase in Ca^2+^ release by the IP_3_R. There is evidence [45] that H_2_O_2_ is able to sensitize the IP_3_R to various stimuli in cultured endothelial cells. This view is supported to a certain extent by our finding that inhibiting PLC produced a significantly greater reduction in the response to carbachol than inhibiting elements of the ROS generating pathway (see Fig. 3).

### 4.3 Reactive oxygen species mediated signalling

It is apparent that H_2_O_2_ generated from the interaction between arachidonic acid and CYP 450 2C9 is important for potentiating the release of Ca^2+^ from stores, since catalase alone, rather that superoxide dismutase reduces effects of carbachol.

The question of whether the Fenton reaction is required for H_2_O_2_ to have its effects was addressed by using Fe^2+^ chelators. The involvement of hydroxyl radical in endothelium-dependent vasodilation was first proposed as a consequence of finding that desferrioxamine (DFO) inhibited bradykinin-induced vasodilatation of mouse cerebral arterioles [46]. Few subsequent studies have addressed this issue, however, and the limited amount of information available on the effects of iron chelation on the EDH as such indicates that hydroxyl radical is not involved [10], [30]. Nonetheless, in light of reports that DFO attenuated arachidonate-induced vasodilatation and permeability increases in rat cerebral arterioles [17], [47], and of our observation that blocking cPLA_2_ suppressed the responses to carbachol in cremaster arterioles, we examined whether these were also affected by DFO and the iron chelating 3-hydroxypyridin-4-ones CP94 and CP85. These have little direct radical scavenging ability [48], moreover a variety of 3-hydroxypyridin-4-ones of varying membrane permeability have been synthesised which allowed us to compare the effects of high (CP94) and low (CP85) cell-permeability analogues [49].

If hydroxyl radical rather than H_2_O_2_ is sensitising the IP_3_R, it would be predicted that CP94 and DFO but not CP85 should inhibit carbachol-induced Ca^2+^ release, since hydroxyl radical, which immediately reacts with other molecules in its vicinity as it is formed, is unlikely to cross the cell membrane [50]. This is what we observed. Although the enormous reactivity of hydroxyl radical would appear to prevent it from mediating signal transduction, as this would require spatially discrete and reversible interactions, there are mechanisms by which this could occur. For example, hydroxyl anion is largely scavenged by ascorbate to yield dehydroascorbate (see [50]), which has been proposed to be transported into the lumen of the ER where it acts to promote the oxidation of protein thiols [51]. This might be of particular relevance to Ca^2+^ release. Notably, it was shown that treatment of aortic EC with H_2_O_2_ leads to glutathionylation of the IP_3_R1 and an increase in its sensitivity to [Ca^2+^] [45], and it was proposed that this might be due to its interaction with ERp44, an ER-resident protein which inhibits the IP_3_R1 by binding to it in a redox-sensitive manner, such that oxidation of cysteine residues on the intraluminal LV3 domain of the IP_3_R1 prevents binding and therefore enhances channel opening [52]. We speculate that such a mechanism could provide a feasible explanation for how the non-localised production of hydroxyl anion within the cytoplasm might result in a localised and reversible effect on signal transduction, in this case an increase in Ca^2+^ release.

Although CP94 profoundly inhibited carbachol-induced endothelial Ca^2+^ release, the cell impermeant CP85 and DFO had no effect (Fig. 6A). In the presence of extracellular Ca^2+^, however, all the chelators reduced the response, which indicates that the hydroxyl radical formed outside the cell membrane is involved in the opening of the K_Ca_ channels (Figs. 6B and 6C), which is evidence for H_2_O_2_ crossing the plasmalemma. There is evidence that several ROS-sensitive Ca^2+^ - permeable channels, including TRPC3, TRPC4 and TRPM2, can contribute to Ca^2+^ influx in EC during the response to muscarinic agonists [27], [53]. Of these, to our knowledge a role for hydroxyl radical as a channel activator has only been reported for TRPM2, although this was shown to involve an action on intracellular cysteine residues [54].

### 4.4 IL-1β and EDH

The ability of IL-1β to promote EDH has not, to our knowledge, been reported previously, although it has been reported that this cytokine caused dilatation of new-born pig pial arterioles *via* a prostanoid- and cyclic nucleotide-dependent-mechanism [55]. The effects of IL-1β were prevented when it was co-applied with the NADPH-oxidase assembly inhibitor apocynin and the PKC blocker Ro31-8220, consistent with previous evidence that Il-1β activates increases superoxide production and activates NADPH oxidase in EC [23], [56], [57]. The effect of IL-1β on the carbachol-induced EDHR in cremaster arterioles demonstrated several parallels with its effect on the bradykinin-induced permeability increase of rat pial venules described by Woodfin et al. [23] including a similar rapid induction and a sensitivity to ROS scavenging, apocynin and PKC blockade, and we speculate that activation of NADPH oxidase may constitute a widespread mechanism by which IL-1β can acutely regulate EC [Ca^2+^]_i_ in the vascular endothelium, and may be one of the factors that are involved in septic shock.

## 5 Conclusions

We speculate that arteries/arterioles may fall into three categories with regard to the involvement of H_2_O_2_ in EDH. In one group, as evidenced by the lack of effect of catalase, H_2_O_2_ is probably not involved in this response [4], although such a role has not been exhaustively examined for every stimulus which triggers EDH in these preparations. In another group, which notably includes coronary arteries, H_2_O_2_ itself seems to act as an important EDHF [6], [7], [10], [11]. The reports of Griffith and colleagues [19], [20] pointed to the existence of a third group of arteries in which H_2_O_2_ facilitates EDH, and focussed attention on IP_3_R-mediated Ca^2+^ release in EC as its likely target. Our results now provide a more detailed picture of how H_2_O_2_ acts to promote EDH in the type of arteriole that falls into this category. We find that very small changes in the EC [H_2_O_2_] around the basal level can strongly modulate the EDHR via an effect on Ca^2+^ release. The data demonstrate that EDH triggered by muscarinic receptor activation is greatly facilitated by ROS which are produced when arachidonic acid synthesised by cPLA_2_ is metabolised by CYP450 2C9. A short pre-treatment with IL-1β is able to further enhance carbachol-induced EDH. An increase in EC Ca^2+^ release, which would be expected to potentiate SOCE, and thereby also facilitate EC Ca^2+^ influx, is implicated in these responses. The effects of CP94 and CP85 indicate that hydroxyl radical plays an important role in this facilitation of Ca^2+^ release, and also demonstrate that ROS also promote EC Ca^2+^ influx during EDH.

A limitation of our results is that we could not record changes in [ROS] in the endothelium of these arterioles, possibly because these were very small. We were therefore unable to determine whether muscarinic receptor activation was increasing EC [ROS], or whether basal ROS levels were sufficient to facilitate Ca^2+^ release. PLA_2_ has, however, been shown to be activated by increases in EC [Ca^2+^]_i_, *e.g.* [58] so that it is plausible that PLA_2_-dependent ROS production would occur upon the application of carbachol.

## Author contributions

James Chidgey: Conception and design of experiments; collection, analysis and interpretation of data.

Paul Fraser: Conception and design of experiments; analysis and interpretation of data, critical revision of the article.

Philip Aaronson: Conception and design of experiments; analysis and interpretation of data: drafting and revising the article.

## Conflict of interest

None declared.

## Footnotes

Appendix A Supplementary data associated with this article can be found in the online version at 10.1016/j.freeradbiomed.2016.06.010.
