# Supplementary material for: Reactive oxygen species facilitate the EDH response in arterioles by potentiating intracellular endothelial Ca2+ release
Source: Free Radic Biol Med. 2016 Aug;97:274–84. doi: 10.1016/j.freeradbiomed.2016.06.010 (PMC5005039; doi:10.1016/j.freeradbiomed.2016.06.010)
Supplement: Supplementary file 1 — Supplementary material [file mmc1.pdf]

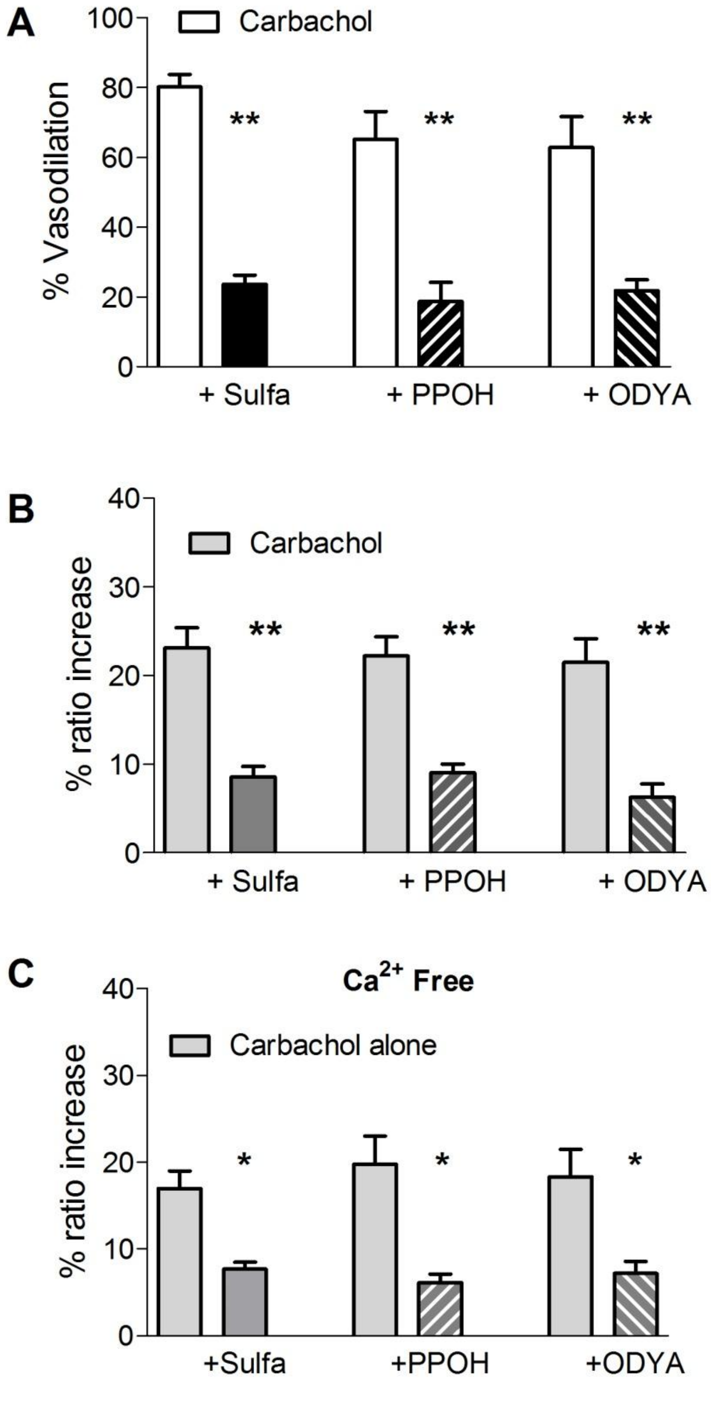

Supplementary Figure 1. Effects of CYP2C9 inhibitors on carbachol-induced responses

(A) Effects of sulfaphenazole (10 μM), PPOH (10 μM) and ODYA (50 μM) on the carbachol induced rise in EC $[Ca^{2+}]_i$ and (B) the associated carbachol-induced vasodilation (n=5 for each condition). (C) These inhibitors were also tested on the carbachol-induced rise in endothelial

$[Ca^{2+}]_i$ in Ca^{2+}-free PSS containing 0.2 mM EGTA. All paired these were paired experiments with 5 to 8 vessels, one animal each; p<0.05 indicated by asterisks.

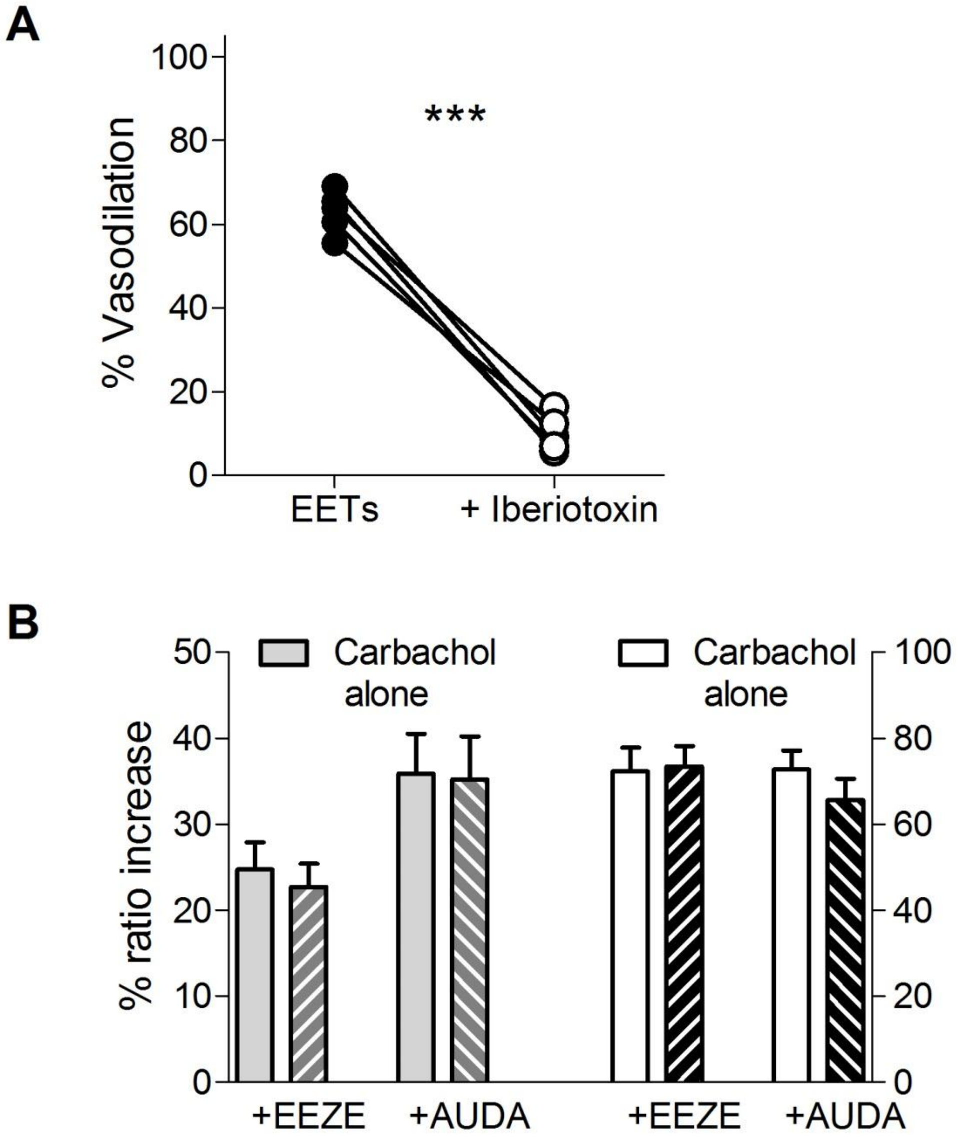

Supplementary Figure 2. Lack of a role for EETs in carbachol-induced EDH.

(A) Application of 11,12 EET (1 μM) resulted in a profound vasodilatation, but (B) neither the EETs receptor blocker, EEZE (10 μM), nor the inhibitor of the enzyme that catabolises EETs, AUDA (10 μM), affected the carbachol-induced vasodilatation (right) or the rise in endothelial $[Ca^{2+}]_i$ (left). 5 vessels from 5 animals; p<0.001 indicated by ***.

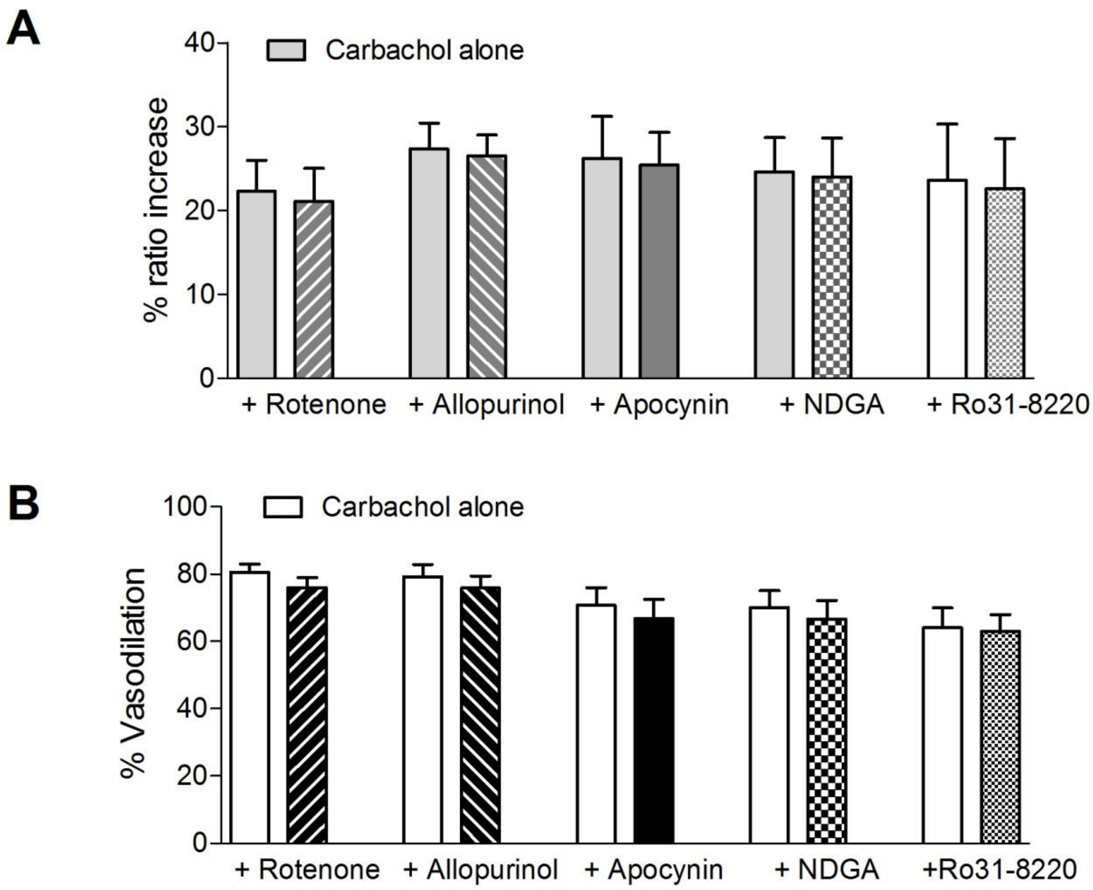

Supplementary Figure 3. Inhibition of several ROS-generating enzymes had no effect on responses to carbachol.

There was no effect when allopurinol (100 µM), apocynin (1 µM), rotenone (10 µM) and NDGA (50 µM) were used to block xanthine oxidase, NADPH oxidase, mitochondrial complex I and lipoxygenases

respectively, on the carbachol-induced rise in (A) the calcium or (B) on the carbachol-induced vasodilation. Normal calcium-containing PSS on 5 vessels from 5 animals for each condition.
